# Glucocorticoid and mineralocorticoid production in hormonally silent adrenocortical tumor tissue in dogs

**DOI:** 10.1093/jvimsj/aalaf087

**Published:** 2026-02-02

**Authors:** Kirsten L van Bokhorst, Marit F van den Berg, Hans S Kooistra, Monique E van Wolferen, Elpetra P M Timmermans-Sprang, Andrea Corsini, Stefania Golinelli, Nicole Bechmann, Mirko Peitzsch, Sara Galac

**Affiliations:** Department of Clinical Sciences, Faculty of Veterinary Medicine, Utrecht University, 3584 CM Utrecht, The Netherlands; IVC Evidensia, 2992 LC Barendrecht, The Netherlands; Department of Clinical Sciences, Faculty of Veterinary Medicine, Utrecht University, 3584 CM Utrecht, The Netherlands; Department of Clinical Sciences, Faculty of Veterinary Medicine, Utrecht University, 3584 CM Utrecht, The Netherlands; Department of Clinical Sciences, Faculty of Veterinary Medicine, Utrecht University, 3584 CM Utrecht, The Netherlands; Department of Clinical Sciences, Faculty of Veterinary Medicine, Utrecht University, 3584 CM Utrecht, The Netherlands; Department of Veterinary Science, University of Parma, 43100 Parma PR, Italy; Department of Veterinary Medical Sciences, University of Bologna, 40064 Ozzano dell'Emilia BO, Italy; Institute for Clinical Chemistry and Laboratory Medicine, University Hospital and Faculty of Medicine Carl Gustav Carus, Technische Universität Dresden, 01307 Dresden, Germany; Institute for Clinical Chemistry and Laboratory Medicine, University Hospital and Faculty of Medicine Carl Gustav Carus, Technische Universität Dresden, 01307 Dresden, Germany; Department of Clinical Sciences, Faculty of Veterinary Medicine, Utrecht University, 3584 CM Utrecht, The Netherlands

**Keywords:** canine, adrenal incidentaloma, hyperadrenocorticism, Cushing’s

## Abstract

**Background:**

No consensus exists regarding the monitoring and therapeutic approach to adrenal tumors (ATs) discovered incidentally by diagnostic imaging, when standard endocrine testing yields negative results.

**Hypothesis/Objectives:**

To evaluate tissue concentrations of adrenocortical steroids in hormonally silent adrenocortical tumors (SATs) in dogs.

**Animals:**

Fourteen dogs with SATs (12 unilateral, 2 bilateral), 11 dogs with cortisol-secreting adrenocortical tumors (cs-ACTs) and 10 healthy dogs.

**Methods:**

Observational study. Diagnosis of SAT was based on finding an AT on diagnostic imaging, negative endocrine function tests, and histopathological and immunohistochemical confirmation. Adrenocortical steroid tissue concentrations were measured by liquid chromatography with tandem mass spectrometry and compared between SATs, cs-ACTs, and normal adrenals (NAs).

**Results:**

Hormonally silent adrenocortical tumors exhibited higher median tissue cortisol (3.62 ng/mg, range 0.05–18.1) and 21-deoxycortisol (0.08 ng/mg, range 0.00–0.44) concentrations than NAs (cortisol 0.38 ng/mg, range 0.01–1.90; 21-deoxycortisol 0.01 ng/mg, range 0.00–0.03; *P* = .04 and *P* = .001, respectively), and these concentrations were not significantly different between SATs and cs-ACTs. Furthermore, SATs’ median tissue concentrations of mineralocorticoid precursors corticosterone (2.15 ng/mg, range 0.01–14.1) and 18-OH-corticosterone (0.70 ng/mg, range 0.00–4.89) were higher than in NAs (respectively 0.19 ng/mg [range 0.14–0.54] and 0.05 ng/mg [range 0.01–0.33]; both *P* = .01) and not different when compared to cs-ACTs.

**Conclusions and clinical importance:**

This study on tissue metabolomics in ATs in dogs demonstrates comparable tissue concentrations of specific glucocorticoids and mineralocorticoids in SATs and cs-ACTs. This implies that some SATs are not hormonally silent, prompting further studies on diagnostics, treatment, and monitoring recommendations.

## Introduction

Adrenal tumors (ATs) are diagnosed with increasing frequency in dogs due to improved availability and quality of diagnostic imaging.^1–3^ If such ATs are identified incidentally during imaging for conditions not related to suspicion of adrenal disease, these tumors are referred to as adrenal incidentalomas (AIs).^1–5^ Based on re-evaluation of clinical signs and endocrine function tests, hypersecretion of hormones from these ATs can be demonstrated, with adrenocortical cortisol (ie, naturally occurring hypercortisolism, HC) and medullary catecholamines (ie, pheochromocytoma) being the most common ones in dogs. If results of endocrine function tests in dogs with AIs are negative, these tumors are defined as non-functional or hormonally silent.^1,6^ For functional or potentially malignant ATs, adrenalectomy is the treatment of choice to eliminate clinical signs of hormone excess and prevent tumor growth and metastasis.^7–14^ Adrenal tumors without hormonal activity or clear evidence of malignancy, however, pose a therapeutic dilemma as currently limited evidence is available about their biological behavior.

In human medicine, an approach to AIs involves the triple testing strategy. Next to tumor diameter and computed tomography (CT) characteristics, urinary steroid metabolomic profiling is an important step in evaluating malignancy and indication for surgical removal of an AI.^15,16^ Metabolomic profiling is a way to study low-molecular weight molecules through biofluids or tissue samples and as such, steroid metabolomics involves analysis of different steroid hormones, precursors, and metabolites.^17–20^ In humans, glucocorticoid and androgen metabolite excess is a characteristic feature of adrenocortical carcinoma (ACC) and this is detected with higher sensitivity through 24-h urinary steroid profiling compared to routine biochemistry.^20,21^ In addition, steroid profiling detects significant steroid precursor excess in 27% of ACC that had been classified as hormonally inactive based on routine biochemical work-up.^21^

Besides analysis of biofluids including urine, metabolomic profiling can be used to analyze alterations in tumor tissue.^17^ There is a distinct catecholamine and tricarboxylic acid cycle metabolite profile in pheochromocytoma tissue in dogs.^22^ Until now, no studies have been performed using metabolomic profiling in adrenocortical tumors (ACTs) in dogs. It is hypothesized that silent adrenocortical tumors (SATs) in dogs might show individual variation in adrenocortical steroid concentrations, including steroids not typically assessed in standard endocrine function tests. Therefore, the current study objective was to analyze adrenocortical steroid tissue concentrations in SATs in dogs and compare these with those in both cortisol-secreting ACTs (cs-ACTs), and normal adrenals (NAs).

## Materials and methods

### Cohort and sample selection

Analyses were performed on ATs collected at 5 European specialist centers, archived in a biobank. Adrenal glands were considered tumorous if a distinct mass was described in either gland, particularly when the mass was heterogenous or invading adjacent vasculature, or if an adrenal gland significantly exceeded normal size as known for different body weight categories.^23,24^ Maximum tumor dimension was recorded for each dog based on imaging techniques (ie, abdominal ultrasonographic examination, CT), as well as evidence for metastases or vascular invasion. In all dogs with an AT, endocrine function tests were performed to assess hormonal hyperactivity, ie, HC, pheochromocytoma, and, specifically in cases with unexplained systemic hypertension or hypokalemia, hyperaldosteronism.^6,25–27^ Cut-off levels for each assay were applied according to the respective laboratory. Diagnosis of cs-ACT was based on clinical signs and abnormal endocrine function tests for HC, performed and interpreted as described previously.^27^ Diagnosis of SAT was based on the presence of an adrenal mass, absence of overt clinical and clinicopathological abnormalities suggestive of adrenal hormone hypersecretion and negative endocrine function tests.^6^

After adrenalectomy, processing and storage of adrenal specimens followed a standardized protocol that is applied across all participating centers. Representative pieces of AT tissue were either snap-frozen in liquid nitrogen or initially preserved in RNAlater stabilization solution (Invitrogen, ThermoFisher Scientific, Breda, the Netherlands) and then stored at –70°C until further processing. Adrenocortical tumor origin was confirmed through histopathology and negative immunohistochemistry of medullary markers chromogranin A (CHGA) and synaptophysin, as described previously.^28^

Normal adrenal glands were obtained from healthy experimental dogs euthanized for reasons unrelated to the present study, which was approved by the institution’s ethical committee. After resection, fat surrounding NAs was carefully dissected, and entire NAs were immediately snap-frozen and stored at –70°C.

### Sample preparation procedure

Tissue samples for adrenocortical steroid measurements were prepared as described previously using the cell disruption buffer of the Paris Kit (AM1921, ThermoFisher Scientific).^22,29^ In NAs, RNA of the same sample specimens was isolated using the Paris Kit following manufacturer instructions (ThermoFisher Scientific).

### Adrenocortical steroids measurements

**Figure 1:**
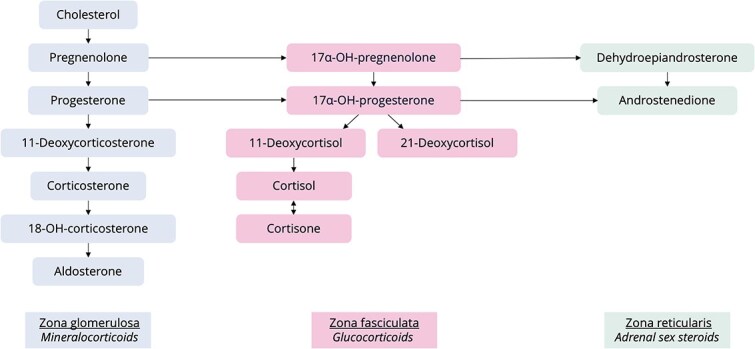
Schematic overview of steroidogenesis in different zones of the adrenal cortex.

Tissue concentrations of adrenocortical steroids (Figure 1) were determined by liquid chromatography tandem mass spectrometry as described previously.^29,30^ Concentrations were calculated relative to tissue sample weight as measured for each sample.

### Normalization of cortical content in normal adrenal glands

Results of NAs were corrected for differences in composition (ie, percentage of adrenal cortex and medulla) as described previously.^22^ Briefly, reverse transcription-quantitative PCR analyses were performed to determine and compare the expression levels of adrenocortical markers steroidogenic acute regulatory protein (*STAR*), cytochrome P450 family 17 subfamily A member 1 (*CYP17A1*), and cytochrome P450 family 11 subfamily B (*CYP11B*), and of adrenomedullary markers *CHGA*, phenylethanolamine-N-methyltransferase (*PNMT*), and tyrosine hydroxylase (*TH*). Calculation of normalized relative mRNA expression levels of the target genes was performed by the 2^–ΔΔCt^ method.^22,31^ The percentage of adrenal cortex was estimated as follows: ([2^–ΔΔCt,STAR^ + 2^–ΔΔCt,CYP17A1^ + 2^–ΔΔCt,CYP11B^]/([2^–ΔΔCt,STAR^ + 2^–ΔΔCt,CYP17A1^ + 2^–ΔΔCt,CYP11B^] + [2^–ΔΔCt,CHGA^ + 2^–ΔΔCt,PNMT^ + 2^–ΔΔCt,TH^])) x 100%. Based on this, adrenocortical steroids tissue concentrations in normal adrenal glands were corrected so that a value corresponding with 100% cortex was used to compare SATs, cs-ACTs, and NAs.

### Statistical analysis

No formal power analysis was performed to determine the optimal sample size, as prior data, including clinically relevant differences in steroid concentrations necessary for this calculation, were not available. Statistical analysis was performed using commercial statistical software (SPSS 27.0.1.0 for Windows, SPSS). The Q–Q plots and Shapiro–Wilk *W*-test were used to assess normality of the data. Non-normally distributed maximum AT dimensions were compared between SAT and cs-ACT groups using Mann–Whitney U tests. Among the three groups (ie, SAT, cs-ACT, and NA), the non-normally distributed tissue steroid concentrations were compared using the Kruskal–Wallis test. If the overall test was significant, post-hoc pairwise comparisons were performed using Dunn-Bonferroni tests to assess differences between groups. For all statistical analyses, *P* < .05 was considered significant.

## Results

### Study group

Hormonally silent adrenocortical tumors (*n* = 16) originated from 12 dogs that underwent unilateral adrenalectomy (*n* = 6 left, *n* = 6 right) and 2 dogs that underwent bilateral adrenalectomy. Eight dogs were female neutered and 6 were male neutered. Breeds included 3 Shih Tzus, 3 mixed breeds, 2 Jack Russell terriers, 2 Stabyhouns, and 1 each of Beagle, French Bulldog, Golden retriever, and West Highland White terrier. Mean age was 10.8 years (SD 2.0 years) and median body weight 8.3 kg (range 5.7–27.8 kg). Indications for abdominal imaging and results of endocrine function tests per individual are shown in Table S1. Endocrine function tests performed to evaluate for HC were the low-dose dexamethasone suppression test (LDDST, 7/14 dogs), high-dose dexamethasone suppression test (1/14 dogs), and urinary corticoid-to-creatinine ratios (6/14 dogs, combined with an oral high-dose dexamethasone suppression test in 4/6 dogs). Endogenous ACTH concentrations were measured in 12/14 dogs with SATs. Plasma aldosterone concentrations and plasma renin activity were measured in respectively 4/14 and 2/14 dogs. Pheochromocytoma was excluded by measurement of plasma metanephrines in 9/14 dogs with SATs and cytological evaluation of a fine-needle aspiration confirmed adrenocortical origin in 2/14 dogs. Cortical origin of SATs was confirmed by histopathology in all dogs. In 2 dogs with SATs, one with immune-mediated hemolytic anemia and one with lower urinary tract disease, AT sizes had been monitored for 7 months and 4.5 years, respectively, and adrenalectomy was performed because of an increase in size (0.9–2.1 cm and 1.6–2.5 cm, respectively).

Cortisol-secreting adrenocortical tumors originated from 11 dogs, which all underwent unilateral adrenalectomy. Of these dogs, 8 were female (6 neutered, 2 intact) and 3 were male (2 neutered, 1 intact). Breeds included 2 Jack Russell terriers and 1 of each of Maltese, Labrador retriever, American Staffordshire terrier, Dachshund, Standard Schnauzer, Weimaraner, Basenji, English bulldog, and mixed breed. Mean age was 9.1 years (SD 1.5 years) and median body weight 10.8 kg (range 5.1–43.6 kg). Results of endocrine function tests per individual are shown in Table S1.

Maximum AT dimension did not differ significantly between SAT (median 2.6 cm, range 1.4–3.7 cm) and cs-ACT (median 3.1 cm, range 1.7–7.0 cm; *P* = .06). A histopathologic diagnosis of adenoma, carcinoma, and unspecified ACT was made in respectively 3/16, 5/16, and 8/16 SATs. No metastases were reported for dogs with SATs. One dog with a cs-ACT had a liver nodule suspected of being metastatic, but histopathological examination did not reveal ACC metastases. Renal vein invasion was suspected based on CT images in 1 SAT (maximum AT dimension 3.5 cm), whereas vascular invasion was suspected in 2 cs-ACTs (one vena cava, one phrenicoabdominal vein; maximum AT dimension 2.9 and 2.9 cm, respectively).

Normal adrenal glands were obtained from 10 healthy dogs of which 9 were intact females and 1 intact male. Breeds included 9 mixed breeds and 1 Beagle. Mean age was 1.7 years (SD 0.7 years).

### Comparison of tissue adrenocortical steroids in SAT, cs-ACT, and NA

**Figure 2:**
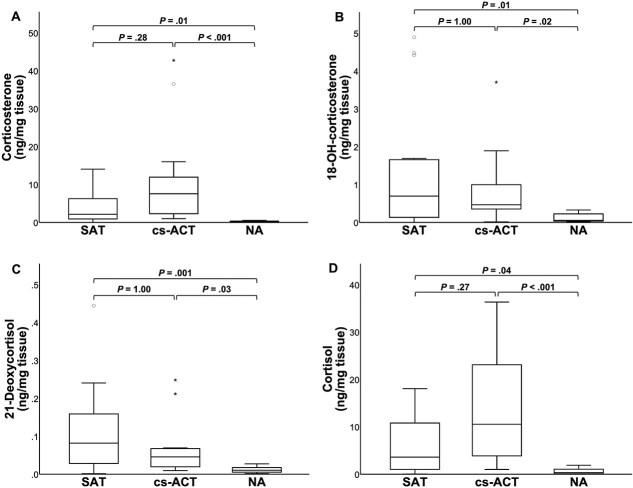
Boxplots of corticosterone (A), 18-OH-corticosterone (B), 21-deoxycortisol (C), and cortisol (D) tissue concentration for SATs, cs-ACTs, and NAs. Boxes represent the interquartile range and lines within the box represent the median. Whiskers represent the range, extending to a maximum of 1.5-times the interquartile range. Circles represent outliers that fall above the third quartile +1.5-times the interquartile range, while asterisks represent extreme outliers exceeding the third quartile +3-times the interquartile range. Abbreviations: SAT = silent adrenocortical tumor; cs-ACT = cortisol-secreting adrenocortical tumor; NA = normal adrenal.

**Figure 3:**
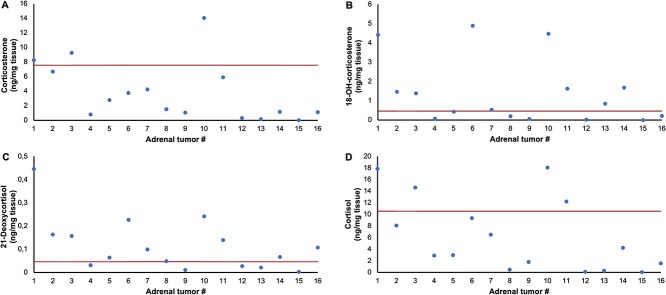
Corticosterone (A), 18-OH-corticosterone (B), 21-deoxycortisol (C), and cortisol (D) tissue concentration for individual hormonally silent adrenocortical tumors. The horizontal line marks the median tissue concentration in cortisol-secreting adrenocortical tumors. #, number as in Table S1.

Results of steroid analysis per group are shown in Table 1 and individual results are shown in Table S2. Median tissue concentrations of corticosterone, 18-OH-corticosterone, 21-deoxycortisol and cortisol were respectively 11-fold (*P* = .01), 13-fold (*P* = .01), 8-fold (*P* = .001) and 10-fold (*P* = .04) higher in SATs compared to NAs, whereas no significant difference existed for these steroid concentrations between SATs and cs-ACTs (Figure 2). Tissue concentrations of corticosterone, 18-OH-corticosterone, 21-deoxycortisol, and cortisol exceeded the highest levels measured in NAs in 13/16, 9/16, 11/16, and 13/16 SATs, respectively. For these steroids, tissue concentrations of individual SATs are presented in Figure 3.

**Table 1:** Absolute steroid tissue concentrations in SATs and cs-ACTs, and steroid tissue concentrations in NAs normalized to cortical content.

	SAT	cs-ACT	NA	*P*-value
Progesterone*	0.87 (0.03–7.52)	2.75 (0.46–24.5)	1.05 (0.30–2.13)	.01
11-Deoxycorticosterone*	0.41 (0.00–5.92)	1.93 (0.24–5.75)	0.33 (0.14–0.78)	.001
Corticosterone*	2.15 (0.01–14.1)	7.57 (1.01–42.8)	0.19 (0.14–0.54)	<.001
18-OH-corticosterone*	0.70 (0.00–4.89)	0.47 (0.01–3.71)	0.05 (0.01–0.33)	.01
11-Dehydrocorticosterone*	2.38 (0.00–5.18)	6.41 (0.36–11.7)	4.61 (2.33–5.88)	.02
**Aldosterone**	0.01 (0.00–0.30)	0.01 (0.00–0.06)	0.01 (0.00–0.63)	.93
**17-OH-progesterone**	0.41 (0.00–2.15)	0.46 (0.10–3.67)	0.40 (0.05–1.08)	.71
21-Deoxycortisol*	0.08 (0.00–0.44)	0.05 (0.01–0.25)	0.01 (0.00–0.03)	.002
11-Deoxycortisol*	1.12 (0.00–4.76)	5.35 (0.44–9.79)	1.35 (0.24–3.67)	.01
Cortisol*	3.62 (0.05–18.1)	10.6 (1.01–36.4)	0.38 (0.01–1.90)	<.001
**18-OH-cortisol**	0.04 (0.00–0.41)	0.04 (0.00–0.13)	0.02 (0.00–0.07)	.32
**18-Oxo-cortisol**	0.00 (0.00–0.02)	0.00 (0.00–0.01)	0.00 (0.00–0.01)	.86
**Cortisone**	1.23 (0.02–4.77)	1.33 (0.15–6.19)	3.10 (1.08–5.04)	.08
Dehydroepiandrosterone*	0.00 (0.00–0.00)	0.02 (0.00–0.09)	0.05 (0.00–0.09)	<.001
Androstenedione*	0.06 (0.00–0.26)	0.32 (0.02–0.95)	0.03 (0.01–0.19)	<.001

Values are expressed as median (range) in ng/mg tissue. Abbreviations: cs-ACT = cortisol-secreting adrenocortical tumor; NA, normal adrenal; SAT = silent adrenocortical tumor. *Statistically significant difference among the three groups (Kruskal–Wallis test).

**Figure 4:**
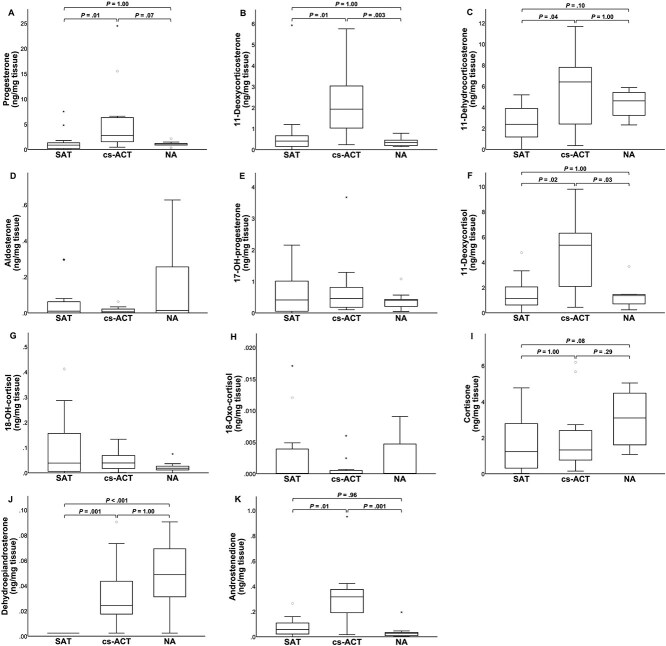
Boxplots of progesterone (A), 11-deoxycorticosterone (B), 11-dehydrocorticosterone (C), aldosterone (D), 17-OH-progesterone (E), 11-deoxycortisol (F), 18-OH-cortisol (G), 18-oxo-cortisol (H), cortisone (I), dehydroepiandrosterone (J), and androstenedione (K) tissue concentration for SATs, cs-ACTs, and NAs. Abbreviations: SAT = silent adrenocortical tumor; cs-ACT = cortisol-secreting adrenocortical tumor; NA = normal adrenal. Boxes represent the interquartile range and lines within the box represent the median. Whiskers represent the range, extending to a maximum of 1.5-times the interquartile range. Circles represent outliers that fall above the third quartile +1.5-times the interquartile range, while asterisks represent extreme outliers exceeding the third quartile +3-times the interquartile range. *P*-values shown only for parameters with significant Kruskal–Wallis tests. Abbreviations: SAT = silent adrenocortical tumor; cs-ACT = cortisol-secreting adrenocortical tumor; NA = normal adrenal.

Of the remaining steroids (Figure 4), median tissue concentrations of 11-deoxycorticosterone, 11-deoxycortisol, and androstenedione were 5-fold higher in cs-ACTs compared to SATs (*P* = .01, *P* = .02, and *P* = .01, respectively) and respectively 6-, 4-, and 11-fold higher compared to NAs (*P* = .003, *P* = .03, and *P* = .001, respectively). Median progesterone and 11-dehydrocorticosterone tissue concentrations were 3-fold higher in cs-ACTs than in SATs (*P* = .01 and *P* = .04, respectively), whereas no significant differences were found between cs-ACTs and NAs and between SATs and NAs. Median dehydroepiandrosterone tissue concentrations were 10-fold higher in cs-ACTs and 20-fold higher in NAs compared to SATs (*P* = .001 and *P* < .001, respectively). No significant differences were found among groups for aldosterone, 17-OH-progesterone, 18-OH-cortisol, 18-Oxo-cortisol, and cortisone.

Cortisol was the predominant steroid in the majority of ATs, ie, in 11/16 SATs and 9/11 cs-ACTs. Of the remaining ATs, 11-dehydrocorticosterone was predominant in 3/16 SATs, progesterone in 1/16 SATs, 11-deoxycortisol in 1/16 SATs, and corticosterone in 2/11 cs-ACTs (Table S2).

In the dog presented because of polyuria/polydipsia, intermittent hyporexia, and diarrhea, with suppressed endogenous ACTH despite negative LDDST results, the SAT (#15) tissue concentrations of all steroid metabolites were on the low end of the group range. In another dog presented because of polyuria/polydipsia with mild hypokalemia and suppressed PRA and aldosterone, the SAT (#12) tissue concentrations of 11-deoxycorticosterone and progesterone were the highest of the SAT group, whereas corticosterone, 18-OH-corticosterone, and aldosterone tissue concentrations were on the low end of the group range.

## Discussion

This study utilizes steroid metabolomic profiling in ACTs in dogs and demonstrates higher tissue concentrations of specific mineralocorticoid and glucocorticoid steroids in SATs, in concentrations comparable to those in cs-ACTs. This suggests that some SATs are not hormonally silent despite negative endocrine function test results.

In line with our findings, a recent study demonstrated no significant difference in protein expression of steroidogenic enzymes cytochrome P450 family 11 subfamily A member 1 (CYP11A1), CYP11B, CYP17, and 3-beta-hydroxysteroid dehydrogenase type 2 between non-functional and functional ACTs.^32^ Overproduction of adrenal sex steroids including dehydroepiandrosterone, androstenedione, and precursor 17-OH-progesterone, is reported in dogs suspected of HC despite negative endocrine function tests.^32–37^ In our cohort of dogs, plasma concentrations of these adrenal sex steroids were not measured as part of the diagnostic work-up. However, excessive adrenal sex steroid hormone effects seem unlikely based on their low tissue concentrations, indicating that at tissue level, our cohort of SATs was not compatible with adrenal sex steroid producing tumors. The dog with suppressed endogenous ACTH concentration despite normal LDDST results does not seem to fit the scenario of steroid precursor excess either, as tissue steroid concentrations were low in this specific SAT (#15). In addition to hyperaldosteronism, clinical signs of polyuria/polydipsia, systemic hypertension, and weakness secondary to hypokalemia have been reported due to excessive secretion of mineralocorticoid precursors including deoxycorticosterone.^38,39^ The dog with mild hypokalemia, suppressed plasma aldosterone, and high deoxycorticosterone and progesterone tissue concentrations (SAT #12) may be an example of this scenario. In the current study, pre- and post-ACTH stimulation cortisol concentrations were not used to exclude HC in SAT cases, as the test has poor sensitivity in adrenal-dependent HC.^27,40^ However, the relevant tissue concentrations of corticosterone, 18-OH-corticosterone, 21-deoxycortisol, and cortisol in SATs, and the latter individual dog (SAT #12), might indicate that these mineralocorticoids and glucocorticoids should be included in further studies on the whole steroid metabolome in SATs, including pre- and post-ACTH stimulation analysis.

In the present study, the diagnosis of SAT was based on absence of clinical signs of adrenal disease and negative standard endocrine function tests. When analyzing the initial reason for diagnostic imaging, some of these dogs presented with polyuria/polydipsia or polyphagia, but none had overt clinical signs of HC (ie, combinations of polyuria/polydipsia with polyphagia, alopecia, or a pot-bellied appearance^27,41^), and endocrine function tests were negative for cortisol excess. However, even with negative endocrine function tests, it is challenging to rule out a relationship between the AT and clinical signs with full certainty. The term sub-diagnostic HC is currently used to describe dogs with clinical signs of HC but negative endocrine test results.^41^ It is possible that some of the dogs diagnosed with SAT would have developed overt clinical signs with abnormal endocrine function test results over time.^27,41^ The scenario of sub-diagnostic HC raises the hypothesis that steroid metabolite tissue concentrations may be higher specifically in dogs with polyuria/polydipsia. However, when analyzing metabolomic profiles of individual SAT’s, it was observed that also dogs which were truly asymptomatic from an adrenal perspective, eg, because they presented with back pain or immune-mediated disease, can have relevant metabolite tissue concentrations.

Earlier publications on ATs in dogs report an association between malignancy and a diameter exceeding 2 cm, irregular structure and invasive growth.^1–3^ However, these data are based on retrospective imaging studies with relatively small numbers of dogs. Furthermore, malignancy in these studies was based solely on an overall histologic evaluation, whereas specific histologic criteria and scores are now known to be better prognostic indicators for cs-ACTs in dogs.^42^ These criteria have, however, not yet been evaluated in SATs in dogs. In humans, significantly elevated glucocorticoid metabolite excretion has been reported in ACA patients, despite no evidence of hormone excess in the routine biochemical work-up.^21^ This supports the concept of a continuum of hormone secretion in ATs, ranging from increased metabolite excretion to clinically overt disease. Urinary steroid profiling is described as a sensitive tool for quantifying steroid excess in human patients with sub-diagnostic HC or borderline primary hyperaldosteronism.^21^ In addition, differences in glucocorticoid and mineralocorticoid metabolites have been demonstrated between human ACAs and ACCs, with the 11-deoxycortisol metabolite tetrahydro-11-deoxycortisol identified as the most discriminative steroid.^21^ The applicability of steroid metabolomics in dogs with sub-diagnostic HC for evaluation of malignancy of ACTs requires further studies.

Dogs with SAT in this study underwent adrenalectomy based on the risks of potential malignancy, based on imaging characteristics including tumor heterogeneity, potential vascular ingrowth and size, after evaluation by a multidisciplinary team including endocrinologists, radiologists, and surgeons. This approach may have resulted in some selection bias, which is inherent to clinical observational studies like the present one. In two dogs in this study, surgery was only performed after an increase in AT size (133% and 56%, respectively) had been demonstrated over time. In human medicine, surgical excision is suggested if the AT enlarges by more than 20% in maximum diameter, in addition to at least a 5 mm increase in maximum diameter, during 6–12 months.^15^ In veterinary medicine, evidence-based guidelines for approaching SATs are still lacking, and the therapeutic decisions are largely arbitrary. Currently, in SATs not eligible for adrenalectomy, a 3-month interval for monitoring with diagnostic imaging is suggested, which may be extended to 6 months in stable situations.^6^ Irrespective of AT growth, repeated endocrine function testing is indicated if clinical signs of endocrine activity appear.^15^

When unilateral adrenalectomy is elected in dogs with SAT, glucocorticoids are typically not supplemented post-surgery. This approach should be reconsidered in light of the significant glucocorticoid tissue concentrations in SATs, as demonstrated in the present study. Additionally, individual glucocorticoid sensitivity, even at sub-diagnostic plasma concentrations, may contribute to symptomatic withdrawal effects after surgery. A postoperative ACTH stimulation test is a practical approach to assess the need for glucocorticoid supplementation and would be valuable to prospectively study in a larger group of dogs with SAT.^6^ In addition, long-term follow-up data for untreated dogs with SATs would be extremely valuable to evaluate whether overt HC or other endocrine disease will develop, or if these tumors become malignant over time. Furthermore, analysis of unenhanced CT tumor attenuation, contrast CT wash-out rates, imaging texture analysis for heterogenicity and contrast-enhanced ultrasonography could all be important studies in dogs with SATs, as these are valuable components in the evaluation for malignancy in human patients.^15,16,20,43,44^

The current study group comprised of smaller-sized dogs (median body weight 8.3 kg) compared to previous studies on the prevalence of AI (median body weights of 28 and 21 kg).^2,3^ All dogs with an SAT in this study were neutered. Whether this reflects a tendency of pet dogs being neutered or a possible relationship with neutering and adrenal neoplasia, eg, via lack of negative feedback of sex steroids on gonadotropic hormone secretion, requires further study.^45^ The lack of difference in maximum tumor dimension between cs-ACTs and SATs in the present cohort is in line with a previous study.^32^

A drawback of this study is that the steroid metabolome analysis was performed in vitro. It remains to be determined whether tissue steroid metabolome mirrors that in urine or plasma and if it can be applied in clinical practice. Another limitation of the study is the relatively low number of ATs included. Finally, although every effort was made to obtain representative tumor tissue samples, intratumoral heterogeneity cannot be completely excluded. This limitation applies equally to both SATs and cs-ACTs.

In conclusion, this study demonstrated comparable glucocorticoid and mineralocorticoid steroid tissue concentrations in SATs and cs-ACTs, indicating that these SATs seem not as hormonally silent as suggested. These findings prompt further analysis of in vivo metabolomic profiling in dogs with SATs to guide therapeutic decisions and monitoring recommendations.

## Supplementary Material

aalaf087_Supplemental_Files

## Off-label antimicrobial declaration

Authors declare no off-label use of antimicrobials.

## Institutional animal care and use committee or other approval declaration

Authors declare no approval was needed, as judged by the Utrecht University’s DEC (Animal Ethics Committee). Signed informed consent was obtained from all owners before study participation.

## Abbreviations

ACA: adrenocortical adenoma
ACC: adrenocortical carcinoma
ACT: adrenocortical tumor
AI: adrenal incidentaloma
AT: adrenal tumor
CHGA: chromogranin A
Cs-ACT: cortisol-secreting adrenocortical tumor
CT: computed tomography
CYP11A1: cytochrome P450 family 11 subfamily A member 1
CYP11B: cytochrome P450 family 11 subfamily B
CYP17A1: cytochrome P450 family 17 subfamily A member 1
HC: hypercortisolism
HSD3B2: 3-beta-hydroxysteroid dehydrogenase type 2
LDDST: low-dose dexamethasone suppression test
NA: normal adrenal
PNMT: phenylethanolamine-N-methyltransferase
SAT: silent adrenocortical tumor
STAR: steroidogenic acute regulatory protein
TH: tyrosine hydroxylase
