# Supplementary material for: Glucocorticoid and mineralocorticoid production in hormonally silent adrenocortical tumor tissue in dogs
Source: J Vet Intern Med. 2026 Feb 2;40(1):aalaf087. doi: 10.1093/jvimsj/aalaf087 (PMC12862633; doi:10.1093/jvimsj/aalaf087)
Supplement: aalaf087_Supplemental_Files [file aalaf087_supplemental_files.zip › Supplementary_Table_1_new.docx]

**Supplementary Table 1.** Clinical signs and indications for abdominal imaging, endocrine function testing and histopathology results per individual dog diagnosed with an adrenocortical tumor.

*Note:* None of the dogs with SATs had overt clinical or clinicopathologic abnormalities suggestive of adrenal hormone hypersecretion. Metanephrines are reported in nmol/L. LDDST results are reported as basal, 4 and 8 hours post-dexamethasone cortisol concentrations in nmol/L. HDDST results are reported as basal and 4 hours post-dexamethasone cortisol concentrations in nmol/L. UCCR results are reported as basal 1, basal 2 and post-oral high-dose dexamethasone suppression ratio. eACTH results are reported in pg/mL. PRA results are reported in fmol/L/s. Aldosterone results are reported in pmol/L. Potassium result is reported in mmol/L.

| AT # | Clinical signs; indications for abdominal imaging | Lesion side | Catecholamine analysis | Glucocorticoid analysis | Mineralocorticoid analysis | Other | Histopathology |
| --- | --- | --- | --- | --- | --- | --- | --- |
| SAT 1 SAT 2 | Lower urinary tract disease | Left  Right | pMN 2.60^A^ pNMN 2.30^A^ | UCCRs: 3.4, 1.8, 0.6^U^  eACTH: 17^U^ |  |  | ACT  ACT |
| SAT 3  SAT 4 | Acute vomiting and hyporexia, mild polyuria/polydipsia | Left  Right | pMN 0.31^A^ pNMN 2.14^A^ | LDDST: 127, 59, 26^I^  eACTH: 7.91^U^ |  |  | ACT  ACT |
| SAT 5 | Acute vomiting (gastric foreign body) | Left |  | LDDST: 136, N/A, 11^I^ |  |  | ACC |
| SAT 6 | Weight loss, liver mass | Right |  | LDDST: 70.6, <27.6, <27.6^M^ eACTH: 13.40^M^ |  | Cytology: ACT | ACT |
| SAT 7 | Elevated liver enzymes on pre-anesthetic screening for dental procedure | Left | pMN 1.27^A^ pNMN 2.7^A^ | UCCR: 2.2^U^  eACTH: 7^U^ |  |  | ACA |
| SAT 8 | Back pain | Left | pMN 0.68^A^ pNMN 1.62^A^ | LDDST: 36, 11, 17  eACTH: 6.4^A^ |  |  | ACT |
| SAT 9 | Pruritus | Left | pMN 0.93^A^ pNMN 2.91^A^ | eACTH: 10.6^B^ |  |  | ACT |
| SAT 10 | Preoperative screening (ovariohysterectomy) | Right |  | HDDST: 58.2, 8.3^B^  eACTH: 12.8^B^ |  |  | ACT |
| SAT 11 | Immune-mediated hemolytic anemia | Right |  | LDDST: 322.8, <27.6, <27.6^M^ |  | Cytology: ACT | ACC |
| SAT 12 | Polyuria/polydipsia | Left | pMN 0.53^A^ pNMN 1.80^A^ | UCCRs: 6.6, 5.8, 1.5^U^  eACTH: 41^U^ | PRA: <40^J^ Aldosterone: <40^U^ | Potassium 2.9^U^ | ACC |
| SAT 13 | Polyuria/polydipsia, body weight gain | Left |  | UCCRs: 3.5, 3.5, 1.5^U^  eACTH: 37^U^ | PRA: 160^J^  Aldosterone: 70^U^ |  | ACC |
| SAT 14 | Polyuria/polydipsia, dysuria (urolithiasis) | Right | pMN 1.24^A^ pNMN 1.29^A^ | UCCR: 1.4^U^  eACTH: 12^U^ |  |  | ACA |
| SAT 15 | Polyuria/polydipsia, intermittent hyporexia and diarrhea | Right | pMN 0.90^A^ pNMN 2.50^A^ | LDDST: 24-3-2^U^  eACTH: <5^U^ | Aldosterone: 24^U^ |  | ACA |
| SAT 16 | Polyphagia, body weight gain | Right | pMN 0.40^A^ pNMN 0.60^A^ | LDDST: 19.5-<2.8-2.8^I^  UCCRs: 1.5, 1.2, 0.5^U^  eACTH: 119^U^ | Aldosterone: 57^U^ |  | ACC |
| cs-ACT 1 | Polyuria, polydipsia, abdominal distension, tremor hind legs, reduced exercise tolerance | Right |  | LDDST: 90, 91, 108^U^  eACTH: 25^U^ |  |  | ACC |
| cs-ACT 2 | Abdominal distension, hepatomegaly* | Left | pMN <0.3^A^ pNMN 0.9^A^ | LDDST: 100, 166, 218^I^  eACTH: 16^U^ |  |  | ACT |
| cs-ACT 3 | Reduced excercise tolerance, polyuria, polydipsia, polyphagia, distended abdomen, abnormal behavior | Right |  | UCCRs: 9.6, 8.2, 7.5^U^  eACTH: 5^U^ |  |  | ACC |
| cs-ACT 4 | Polyuria, polydipsia, polyphagia, poor hair coat, reduced excercise tolerance | Left |  | LDDST: 106, 112, 109^I^ eACTH: <5^U^ |  |  | ACC |
| cs-ACT 5 | Polyuria, polydipsia, polyphagia | Right |  | UCCRs: 10, 10, 10^U^ eACTH: <5^U^ |  |  | ACC |
| cs-ACT 6 | Polyuria, polydipsia, polyphagia, lethargy, body weight gain | Left |  | UCCRs: 53.4, 48.4, 44.6^U^ eACTH: 5^U^ |  |  | ACA |
| cs-ACT 7 | Polyuria, polydipsia, polyphagia, abdominal distension, symmetrical alopecia | Left |  | ACTH-stimulation test: 110, 1068^I^ LDDST: abnormal** eACTH: 18^U^ |  |  | ACC |
| cs-ACT 8 | Polyuria, polydipsia, polyphagia, poor hair coat, abdominal distension | Left |  | LDDST: abnormal**  eACTH: suppressed** |  |  | ACC |
| cs-ACT 9 | Polyuria, polydipsia, polyphagia, alopecia, muscle atrophy, distended abdomen | Left |  | UCCRs: 20, 24, 20^U^ eACTH: <5^U^ |  |  | ACC |
| cs-ACT 10 | Polyuria, polydipsia, polyphagia, abdominal distension | Right |  | UCCRs: 68.3, 41.6, 50.3^U^ eACTH: 9^U^ |  |  | ACC |
| cs-ACT 11 | Polyuria, polydipsia, polyphagia | Right |  | UCCRs: 15, 19, 32^U^ eACTH: <5^U^ |  |  | ACC |

Abbreviations: AT, adrenal tumor; SAT, silent adrenocortical tumor; cs-ACT, cortisol-secreting adrenocortical tumor; pMN, plasma free metanephrine; pNMN, plasma free normetanefrine; LDDST, low-dose dexamethasone suppression test; HDDST, high-dose dexamethasone suppression test; UCCR, urinary corticoid-to-creatinine ratio; eACTH: endogenous ACTH; PRA, plasma renin activity; ACT, adrenocortical tumor (inconclusive); ACC, adrenocortical carcinoma; ACA, adrenocortical adenoma.

Symbols refer to specific laboratories: ^A^Algemeen Medisch Laboratorium (AML). ^I^IDEXX Laboratories. ^M^MyLav. ^B^Bologna University laboratory. ^U^Universitair Veterinair Diagnostisch Laboratorium (UVDL). ^J^Javadi et al. 2003, reference doi: 10.1136/vr.153.17.521.

*Abdominal distension normalized and skin condition improved after adrenalectomy.

** Exact results were no longer available (cases dating from 2010 and 2013). Patient cs-ACT #7: positive ACTH stimulation test and the diagnostic imaging findings were consistent with adrenal-dependent hypercortisolism. Patient cs-ACT #8: clinical and diagnostic imaging findings consistent with adrenal-dependent hypercortisolism. Recurrence of HC was observed after 8 months; at this stage, metastases in the liver and thorax were visualized and confirmed in liver by cytology. Additionally, UCCR was elevated and eACTH suppressed, confirming clinical suspicion of hypercortisolism.
