# Supplementary material for: Glucocorticoid and mineralocorticoid production in hormonally silent adrenocortical tumor tissue in dogs
Source: J Vet Intern Med. 2026 Feb 2;40(1):aalaf087. doi: 10.1093/jvimsj/aalaf087 (PMC12862633; doi:10.1093/jvimsj/aalaf087)
Supplement: aalaf087_Supplemental_Files [file aalaf087_supplemental_files.zip › Supplementary_Table_2_new.docx]

**Supplementary Table 2.** Adrenocortical steroid concentrations in individual silent adrenocortical tumors, cortisol-secreting adrenocortical tumors and normal adrenal glands.

| AT # | Progesterone | 11-Deoxycorticosterone | Corticosterone | 18-OH-corticosterone | 11-Dehydrocorticosterone | Aldosterone | 17-OH-progesterone | 21-Deoxycortisol | 11-Deoxycortisol | Cortisol | 18-OH-cortisol | 18-Oxo-cortisol | Cortisone | Dehydroepiandrosterone | Androstenedione |
| --- | --- | --- | --- | --- | --- | --- | --- | --- | --- | --- | --- | --- | --- | --- | --- |
| SAT 1 | 1.36 | 0.60 | 8.26 | 4.42 | 4.55 | 0.05 | 1.13 | 0.44 | 2.30 | **17.88** | 0.28 | 0.00 | 2.62 | 0.00 | 0.09 |
| SAT 2 | 1.29 | 0.69 | 6.69 | 1.47 | 3.09 | 0.03 | 0.32 | 0.16 | 0.96 | **8.08** | 0.17 | 0.00 | 2.22 | 0.00 | 0.07 |
| SAT 3 | 0.21 | 0.61 | 9.28 | 1.39 | 5.13 | 0.02 | 0.03 | 0.16 | 1.10 | **14.64** | 0.15 | 0.00 | 1.90 | 0.00 | 0.04 |
| SAT 4 | 0.03 | 0.27 | 0.80 | 0.07 | 0.21 | 0.00 | 0.06 | 0.03 | 1.49 | **2.92** | 0.01 | 0.00 | 0.17 | 0.00 | 0.02 |
| SAT 5 | 1.79 | 0.43 | 2.78 | 0.43 | 1.28 | 0.00 | 0.68 | 0.06 | 1.14 | **3.00** | 0.02 | 0.00 | 0.91 | 0.00 | 0.05 |
| SAT 6 | 4.80 | 1.20 | 3.77 | 4.89 | 4.06 | 0.30 | 2.15 | 0.23 | 4.76 | **9.39** | 0.14 | 0.01 | 2.95 | 0.00 | 0.11 |
| SAT 7 | 0.16 | 0.32 | 4.26 | 0.54 | 5.18 | 0.00 | 0.05 | 0.10 | 1.81 | **6.53** | 0.01 | 0.00 | 1.55 | 0.00 | 0.03 |
| SAT 8 | 0.40 | 0.39 | 1.52 | 0.20 | **1.60** | 0.00 | 0.01 | 0.05 | 0.13 | 0.47 | 0.00 | 0.00 | 0.11 | 0.00 | 0.00 |
| SAT 9 | 0.06 | 0.05 | 1.05 | 0.06 | 0.91 | 0.00 | 0.12 | 0.01 | 0.41 | **1.82** | 0.01 | 0.00 | 0.87 | 0.00 | 0.16 |
| SAT 10 | 0.79 | 0.87 | 14.07 | 4.48 | 1.07 | 0.08 | 0.71 | 0.24 | 3.32 | **18.08** | 0.29 | 0.02 | 0.56 | 0.00 | 0.10 |
| SAT 11 | 0.95 | 0.10 | 5.91 | 1.63 | 2.24 | 0.02 | 2.08 | 0.14 | 0.66 | **12.25** | 0.41 | 0.00 | 3.32 | 0.00 | 0.16 |
| SAT 12 | 7.52 | 5.92 | 0.31 | 0.03 | 1.58 | 0.00 | 1.07 | 0.03 | **1.67** | 0.12 | 0.00 | 0.00 | 0.26 | 0.00 | 0.26 |
| SAT 13 | 0.14 | 0.06 | 0.16 | 0.85 | **3.12** | 0.08 | 0.20 | 0.02 | 0.58 | 0.30 | 0.05 | 0.00 | 4.26 | 0.00 | 0.02 |
| SAT 14 | 0.59 | 0.63 | 1.17 | 1.69 | 3.72 | 0.08 | 0.50 | 0.07 | 2.85 | **4.24** | 0.06 | 0.00 | 4.77 | 0.00 | 0.10 |
| SAT 15 | **1.05** | 0.00 | 0.01 | 0.00 | 0.00 | 0.00 | 0.00 | 0.00 | 0.00 | 0.05 | 0.00 | 0.00 | 0.02 | 0.00 | 0.00 |
| SAT 16 | 1.13 | 0.21 | 1.12 | 0.21 | **2.53** | 0.00 | 0.95 | 0.11 | 0.94 | 1.57 | 0.00 | 0.00 | 0.37 | 0.00 | 0.02 |
| cs-ACT 1 | 0.89 | 0.89 | 7.80 | 1.14 | 4.20 | 0.01 | 0.10 | 0.01 | 1.25 | **8.41** | 0.05 | 0.00 | 1.37 | 0.07 | 0.21 |
| cs-ACT 2 | 1.43 | 1.16 | 7.57 | 0.41 | 3.06 | 0.00 | 0.93 | 0.25 | 8.31 | **10.56** | 0.02 | 0.00 | 2.08 | 0.00 | 0.34 |
| cs-ACT 3 | 0.46 | 0.24 | 1.01 | 0.32 | 0.36 | 0.03 | 0.14 | 0.04 | 0.44 | **1.39** | 0.02 | 0.00 | 0.22 | 0.02 | 0.02 |
| cs-ACT 4 | 6.04 | 5.65 | 42.75 | 3.71 | 6.41 | 0.01 | 0.33 | 0.07 | 5.09 | **36.35** | 0.13 | 0.00 | 0.87 | 0.02 | 0.13 |
| cs-ACT 5 | 1.66 | 3.28 | 2.87 | 0.29 | 7.87 | 0.01 | 0.15 | 0.01 | 9.79 | **3.80** | 0.03 | 0.00 | 6.19 | 0.03 | 0.22 |
| cs-ACT 6 | 2.93 | 0.65 | 5.50 | 0.47 | 8.62 | 0.03 | 3.67 | 0.21 | 5.38 | **25.33** | 0.08 | 0.00 | 5.66 | 0.06 | 0.41 |
| cs-ACT 7 | 2.58 | 1.60 | 7.90 | 0.38 | 7.72 | 0.00 | 0.46 | 0.01 | 6.62 | **15.17** | 0.01 | 0.00 | 2.73 | 0.09 | 0.95 |
| cs-ACT 8 | 2.75 | 1.93 | 16.03 | 0.86 | 6.88 | 0.00 | 0.69 | 0.05 | 5.35 | **20.90** | 0.04 | 0.00 | 1.21 | 0.01 | 0.17 |
| cs-ACT 9 | 24.46 | 2.78 | 1.77 | 0.49 | 1.76 | 0.02 | 0.51 | 0.07 | 2.93 | **3.97** | 0.05 | 0.00 | 0.66 | 0.02 | 0.33 |
| cs-ACT 10 | 15.51 | 2.19 | **1.36** | 0.01 | 1.56 | 0.00 | 1.29 | 0.06 | 1.02 | 1.01 | 0.00 | 0.00 | 0.15 | 0.03 | 0.42 |
| cs-ACT 11 | 6.58 | 5.75 | **36.50** | 1.90 | 11.68 | 0.06 | 0.21 | 0.03 | 5.99 | 29.08 | 0.11 | 0.01 | 1.33 | 0.02 | 0.32 |
| NA 1 | 0.33 | 0.16 | 0.14 | 0.06 | **3.30** | 0.02 | 0.05 | 0.00 | 0.24 | 0.01 | 0.00 | 0.00 | 1.08 | 0.04 | 0.01 |
| NA 2 | 1.12 | 0.33 | 0.15 | 0.05 | **2.33** | 0.02 | 0.40 | 0.01 | 1.44 | 0.24 | 0.01 | 0.00 | 2.30 | 0.07 | 0.05 |
| NA 3 | 1.47 | 0.49 | 0.54 | 0.08 | **4.82** | 0.00 | 0.57 | 0.02 | 1.45 | 1.90 | 0.04 | 0.00 | 3.15 | 0.08 | 0.03 |
| NA 4 | 1.02 | 0.39 | 0.49 | 0.03 | **5.41** | 0.00 | 0.41 | 0.03 | 1.46 | 1.21 | 0.03 | 0.00 | 4.47 | 0.00 | 0.03 |
| NA 5 | 1.21 | 0.34 | 0.17 | 0.27 | **4.50** | 0.26 | 0.31 | 0.01 | 1.28 | 0.51 | 0.07 | 0.01 | 4.62 | 0.00 | 0.03 |
| NA 6 | 1.06 | 0.45 | 0.17 | 0.23 | **5.88** | 0.28 | 0.21 | 0.01 | 1.42 | 0.19 | 0.02 | 0.00 | 3.61 | 0.05 | 0.03 |
| NA 7 | 1.05 | 0.19 | 0.21 | 0.33 | **3.24** | 0.63 | 0.42 | 0.01 | 0.41 | 0.12 | 0.02 | 0.01 | 1.30 | 0.06 | 0.01 |
| NA 8 | 0.30 | 0.14 | 0.15 | 0.01 | **5.54** | 0.01 | 0.08 | 0.00 | 0.70 | 0.13 | 0.00 | 0.00 | 3.06 | 0.04 | 0.01 |
| NA 9 | 0.87 | 0.28 | 0.32 | 0.03 | **2.44** | 0.00 | 0.43 | 0.01 | 0.75 | 1.07 | 0.02 | 0.00 | 1.61 | 0.03 | 0.02 |
| NA 10 | 2.13 | 0.78 | 0.39 | 0.04 | **4.73** | 0.00 | 1.08 | 0.02 | 3.67 | 0.90 | 0.02 | 0.00 | 5.04 | 0.09 | 0.19 |

*Note:* Values are expressed as ng/mg tissue. The predominant steroid of each adrenal tumor or normal adrenal gland is marked in bold. Abbreviations: SAT, silent adrenocortical tumor; cs-ACT, cortisol-secreting adrenocortical tumor; NA, normal adrenal.
